# Distinct immune and inflammatory response patterns contribute to the identification of poor prognosis and advanced clinical characters in bladder cancer patients

**DOI:** 10.3389/fimmu.2022.1008865

**Published:** 2022-10-27

**Authors:** Zhenglin Chang, Rongqi Li, Jinhu Zhang, Lingyue An, Gaoxiang Zhou, Min Lei, Jiwang Deng, Riwei Yang, Zhenfeng Song, Wen Zhong, Defeng Qi, Xiaolu Duan, Shujue Li, Baoqing Sun, Wenqi Wu

**Affiliations:** ^1^ Department of Urology, The Second Affiliated Hospital of Guangzhou Medical University, Guangzhou, Guangdong, China; ^2^ Department of Allergy and Clinical Immunology, Department of Laboratory, National Center for Respiratory Medicine, National Clinical Research Center for Respiratory Disease, State Key Laboratory of Respiratory Disease, Guangzhou Institute of Respiratory Health, The First Affiliated Hospital of Guangzhou Medical University, Guangzhou, China; ^3^ Guangdong Key Laboratory of Urology, The First Affiliated Hospital of Guangzhou Medical University, Guangzhou, China; ^4^ Department of Hepatobiliary Surgery, Foshan Hospital of Traditional Chinese Medical, Foshan, Guangdong, China

**Keywords:** bladder cancer, chemotherapy, immunotherapy, immune and inflammatory characteristics, unsupervised cluster analysis

## Abstract

Due to the molecular heterogeneity, most bladder cancer (BLCA) patients show no pathological responses to immunotherapy and chemotherapy yet suffer from their toxicity. This study identified and validated three distinct and stable molecular clusters of BLCA in cross-platform databases based on personalized immune and inflammatory characteristics. H&E-stained histopathology images confirmed the distinct infiltration of immune and inflammatory cells among clusters. Cluster-A was characterized by a favorable prognosis and low immune and inflammatory infiltration but showed the highest abundance of prognosis-related favorable immune cell and inflammatory activity. Cluster-B featured the worst prognosis and high immune infiltration, but numerous unfavorable immune cells exist. Cluster-C had a favorable prognosis and the highest immune and inflammatory infiltration. Based on machine learning, a highly precise predictive model (immune and inflammatory responses signature, IIRS), including FN1, IL10, MYC, CD247, and TLR2, was developed and validated to identify the high IIRS-score group that had a poor prognosis and advanced clinical characteristics. Compared to other published models, IIRS showed the highest AUC in 5 years of overall survival (OS) and a favorable predictive value in predicting 1- and 3- year OS. Moreover, IIRS showed an excellent performance in predicting immunotherapy and chemotherapy’s response. According to immunohistochemistry and qRT-PCR, IIRS genes were differentially expressed between tumor tissues with corresponding normal or adjacent tissues. Finally, immunohistochemical and H&E-stained analyses were performed on the bladder tissues of 13 BLCA patients to further demonstrate that the IIRS score is a valid substitute for IIR patterns and can contribute to identifying patients with poor clinical and histopathology characteristics. In conclusion, we established a novel IIRS depicting an IIR pattern that could independently predict OS and acts as a highly precise predictive biomarker for advanced clinical characters and the responses to immunotherapy and chemotherapy.

## Introduction

Bladder Cancer (BLCA) has become an increasingly prominent public health issue worldwide due to its high recurrence rate, high metastatic propensity, and peculiar chemo- and radio-resistance (1, 2). Radical cystectomy can perform well in resecting localized tumors, and cisplatin-based neoadjuvant chemotherapy (NAC) remains the most established perioperative option so far. Nevertheless, the survival probability of patients after an operation is extremely low, and nearly 50% of them will ultimately experience the spread of cancer (3, 4). In the past decade, immunotherapy has evolved as one of the most promising advancements, deeply revolutionizing the therapeutic paradigm of BLCA. Factually, some immune checkpoint inhibitors have already been approved in advanced BLCA (5). Unfortunately, most patients show no pathological responses to immunotherapy and chemotherapy yet suffer from their toxicity. The distinct sensitivity to therapy might primarily be due to the molecular and genetic heterogeneity of the tumor microenvironment (TME) in BLCA (1, 3). Hence, there is an urgent need to identify the potential molecular subtype and develop novel and reliable markers to predict prognosis, chemotherapy, and immunotherapy efficacy for BLCA.

The initiation and progression of tumors not only depend on the genetic heterogeneity of malignant cells but also on the tumor microenvironment (TME) (6). The inflammatory milieu has been considered a pivotal aspect of a tumor, affecting various hallmarks of the tumor, including cell proliferation, angiogenesis, invasion, and metastasis (7, 8). Recently, a growing number of clinical and experimental evidences indicated that the acute inflammatory response could prevent the growth and invasion of the tumor, while chronic inflammation can aid the transformation of malignancy (9–11). Moreover, innate immunity can promote tumorigenesis, while adaptive immunity often restrains through immunosurveillance (7, 9, 12). It is worth noting that immune and inflammatory responses (IIR) play distinct roles in different stages of tumor progression, and the balance of IIR is crucial in restricting the development and progression of malignancy. The normal controlled inflammatory response can activate a specific immune response, resulting in healing (13). Conversely, prolonged and dysregulated inflammation is closely related to immune suppression, thus leading to disease progression (13, 14). The immune and inflammatory process is dynamic and changing over time, reflecting the degree of the normal or abnormal responses of the body. Although increasing research has focused on the molecular characteristics of BLCA based on gene expression patterns of immune or inflammation (15–19), there has been no report of prognosis-related classification that combined immune with inflammatory response patterns. Therefore, it is meaningful to identify the distinct IIR patterns and develop a novel prognosis, immunotherapy, and chemotherapy-related signature based on IIR patterns.

The IIR patterns and IIR-based signature (IIRS) were identified for the first time in this study using two cross-platform BLCA data. In addition, bladder tissues and corresponding clinical characteristics of BLCA patients were collected to validate the clinical application potential of the IIRS-score. These findings will unveil unknown molecular subtypes of TME-related immune and inflammatory responses and make current immunotherapy and chemotherapy strategies more efficient, optimizing the chance of response and reducing the overtreatment of non-responders.

## Methods

### Dataset acquisition and preparation

The RNA-Seq data of BLCA with matched clinicopathological features were obtained from TCGA database. The microarray data set GSE32894 with corresponding clinical information was downloaded from the GEO database. IMvigor210 trial, including patients with metastatic urothelial cancer treated with atezolizumab, was obtained from http://research-pub.gene.com/IMvigor210CoreBiologies/. In two data sets, the selection criteria were as follows: (1) have survival data; (2) follow-up ≥ 1 month; (3) pathological diagnosis was BLCA. Finally, 403 BLCA samples of TCGA, 224 of GSE32894, and 348 of IMvigor210 were enrolled in this study.

### Identification of immune and inflammatory phenotype-related genes (IIRGs)

We analyzed six inflammation-related gene sets (M3952, M5932, M6910, M17322, M38152, and M39641) from the GSEA database, representing diverse targets from inflammatory cells, cytokines, pathways, and responses. In addition, immune-related genes were picked from the Tracking Tumor Immunophenotype database. These targets were put into the STRING database, and CytoNCA plug-in was applied to sieve the IIRGs.

### Identification of the IIR-related patterns by consensus clustering

Based on these identified IIRGs, the consensus clustering method was applied to identify novel IIR patterns in TCGA cohort to classify patients. We limited the clustering algorithm to ‘pam’ and performed 100 iterations here. Two GEO cohorts were utilized to verify the clustering stability using the same method. To determine the optimal clustering number, we applied the PCA method to extract the data of the consensus matrix, followed by generating the fitting curve using the ‘ecdf’ method. After calculating the area under the cumulative distribution function (CDF) curve between 0.1 and 0.9, the corresponding K was regarded as the optimal number of clustering according to the minimum area under CDF. The overall survival (OS) among classifications was calculated using the Kaplan-Meier (KM) method. Principal component analysis (PCA) was also utilized to demonstrate expression patterns of IIRGs in different BLCA patients.

### Comparison of tumor microenvironment infiltration patterns

Based on the ESTIMATE algorithm, we generated the immune score, stromal score, and tumor purity. We then generated the inflammatory score based on the ssGSEA algorithm using the identified inflammation-related gene sets. Next, CIBERSORTX was applied to evaluate the relative abundances of multiple immune cell types (20). The influence of immune cell types on survives was determined by classifying the patient’s samples into high or low groups according to relative expression levels of immune cell types. Cases were divided into two groups based on the relative expression levels of immune cell types, and then the prognoses of BLCA patients with different immune cell expression levels were analyzed by the Kaplan–Meier survival curve. Subsequently, the univariate Cox regression was applied to identify the prognostic-related differentially expressed immune cells among clusters.

### Identification of the BLCA-related inflammation activity signature

We established scoring systems to quantify the different inflammatory activity patterns to increase our understanding of cluster-related inflammatory activities. First, 11 signatures of inflammatory activity related to the progression of bladder cancer were selected from the GSEA database (**Supplementary Table 1**). Then, using a univariate Cox regression model, we performed prognostic analysis on the genes in each signature. Next, genes with significant prognoses (p<0.05) were extracted for further analysis. Afterward, PCA was performed to establish inflammatory signatures. Both principal components 1 and 2 were selected as signature scores. Finally, the scores were defined using a method similar to Genomic Grade Index (21, 22):


score=∑(PC1i+PC2i)

Where i is the expression of inflammatory activity-related genes with a significant prognosis among clusters.

### Construction of IIR-based signature (IIRS)

To conduct the quantitative assessment of the IIR pattern of each patient, we constructed a scoring system termed IIRS. First, the “limma” package was utilized to filter the differentially expressed IIRGs (DEIIRGs) among patterns. The optimal IIRS was then established based on the machine learning and Cox regression. We then calculated the IIRS-score (risk-Score) of each patient to predict the prognosis of BLCA and utilized the “survminer” package to determine the optimal cut-off score for the risk. Next, the time-dependent receiver operating characteristic (ROC) curve analysis was conducted to compare the predictive accuracy and other clinicopathological characteristics of IIRS. Moreover, we picked five reported immune or inflammatory signatures to compare the prognostic values (15–19). Finally, the clinical utility was evaluated by decision curve analysis (DCA).

### Gene set enrichment analysis of the IIRS genes

Based on the median expression of IIRS genes, 403 BLCA patients were divided into two groups. First, GSEA was conducted to investigate the potential molecular mechanisms of IIRS genes, setting the “c2.cp.kegg.v7.1.-symbols.gmt” as the reference gene set. The top 10 terms of each IIRS gene were obtained after excluding unrelated signaling pathways such as “acute myeloid leukemia” and “endometrial cancer”.

### Biomarkers for predicting immunotherapy response and chemotherapeutic response

The tumor mutation burden (TMB) of each TCGA-BLCA patient was calculated by Perl script. Then, we divided patients into four groups based on the median cut-offs of IIRS-score and TMB to see if the IIRS-TMB joint diagnosis had a thorough prediction ability. A total of 50 checkpoints were collected from the previously published articles. In addition, IMvigor210 cohorts were included in our study to see if the IIRS could predict clinical response to PD-1 blockers. Moreover, we applied The Cancer Immunome Atlas (TCIA) to detect the immunophenoscore (IPS) of tumor samples, which can predict the clinical response to CTLA-4 and PD-1 blockers. Finally, to provide individualized medication for each BLCA patient, we picked underlying drugs of BLCA in the ‘pRRophetic’ package according to previously published articles, followed by evaluating the half-maximal inhibitory concentration (IC50) of each drug.

### Tumor mutation analysis of DEIIRGs

The single nucleotide variants data of BLCA samples based on the “VarScan” process were obtained from the TCGA database. Mutation data were visualized using the “maftools” package to identify the somatic mutations of the patients with the identified genes of IIRGs.

### Quantitative real-time polymerase chain reaction (qRT-PCR)

Three bladder samples of carcinomas and adjacent normal tissues were obtained from BLCA patients who underwent surgery at Guangzhou Medical University’s Second Affiliated Hospital (GZhmu2.cohort) without preoperative immunotherapy or chemotherapy. RNA extraction and qRT-PCR were conducted as previously described (22, 23). The PCR primers are described in **Supplementary Table 2**. The relative expression of IIRS genes was calculated from three technical replicates using the 2 −^ΔΔCt^ method.

### Hematoxylin and eosin (H&E)

A total of 403 whole-slide H&E-stained histopathology images were obtained from TCGA. Moreover, 14 bladder samples of cancer tissues were obtained from BLCA patients who underwent surgery at Guangzhou Medical University’s First Affiliated Hospital (GZhmu1.cohort) without preoperative immunotherapy or chemotherapy. The corresponding clinical information is displayed in **Supplementary Table 3**. A single tissue was too small to scan and was abandoned for subsequent analysis. All images were observed by a tissue scanner (PathScope 4s, DigiPath, NV, USA) to observe the inflammatory and immune histological changes of bladder tissues. The corresponding score system was performed as previously described (22, 23).

### Immunohistochemistry (IHC)

The primary antibodies: CD247 (1:200), TLR2 (1:200), c-MYC (1:500), IL10 (1:200), and FN1 (1:200) were obtained from Proteintech (Rosemont, IL, USA) and were applied for IHC. IHC was conducted as previously described (22, 23). For each IHC-tissue section, five random visual fields were selected for determination. The relative expressions of IIRS genes were obtained by calculating the means of integrated optical density (MOD) using ImageJ software.

### Statistical analyses

R and IBM SPSS Statistics were used to conduct statistical analyses. The KM analysis was conducted using the log-rank test. The Hazard Ratio (HR) and 95% confidence interval (CI) were generated using KM and Cox regression. Statistical significance of the comparison between two groups for continuous variables and categorical variables was estimated by Student’s T-test or Mann-Whitney-Wilcoxon test and Chi-square test or Fisher’s exact tests, respectively. The existence of a correlation between variables was accessed by Spearman correlation analysis. The statistical significance of PCR and IHC were assessed by t-tests or one-way ANOVA with LSD *post hoc* comparisons or Dunnett’s T3 *post hoc* tests. A two-tailed P value less than 0.05 was considered statistically significant.

## Results

### Collection of IIRGs

**Figure 1:**
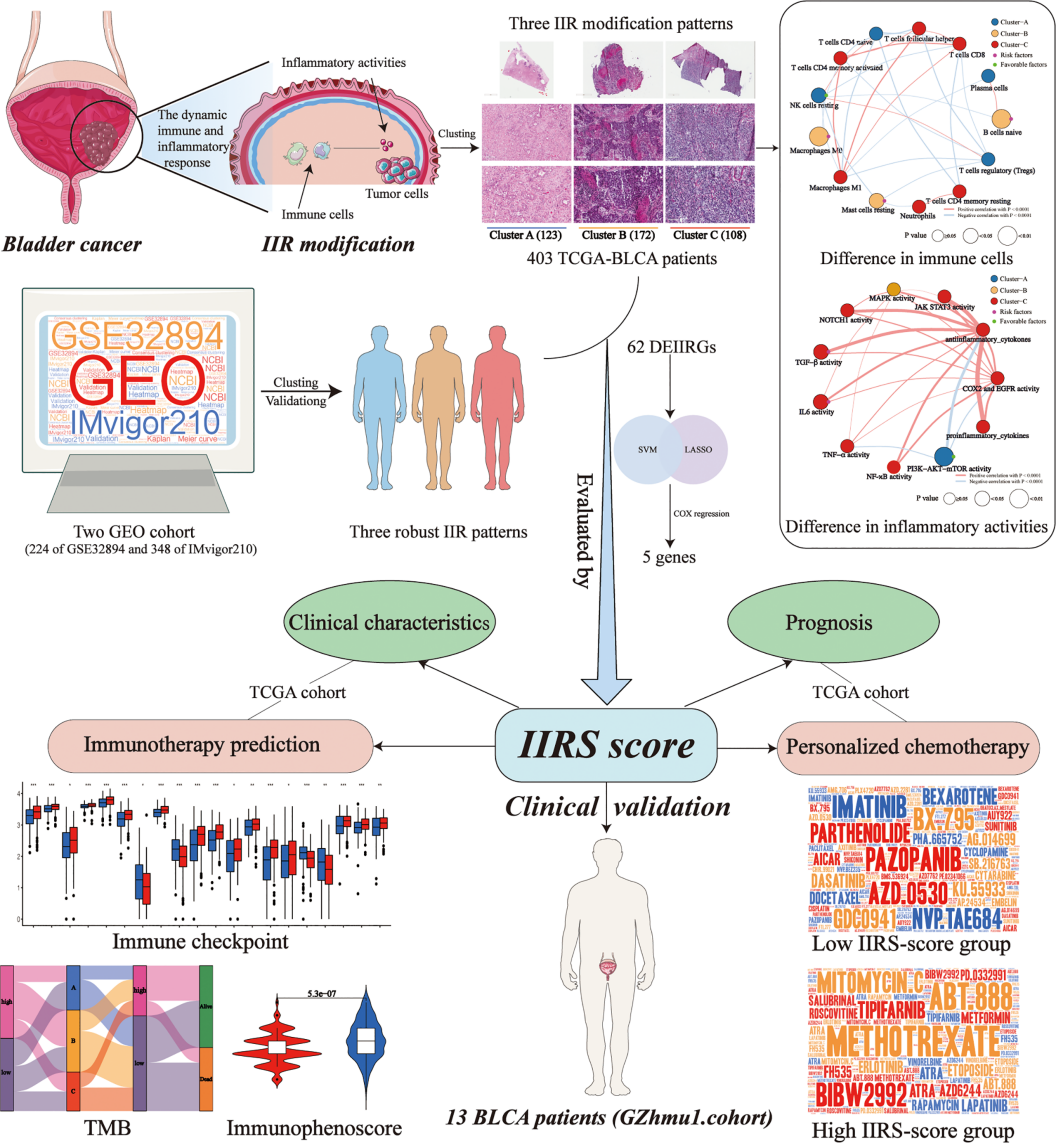
The workflow of designed analysis.

The workflow is shown in **Figure 1**. 1193 inflammatory genes and 178 immune genes were enrolled into our analysis (**Supplementary Figures 1A, B**). After filtering, a total of 85 IIRGs were selected (**Supplementary Figures 1C-F**, **Supplementary Table 4**).

### Three distant IIR Clusters with different clinical outcomes of BLCA

**Figure 2:**
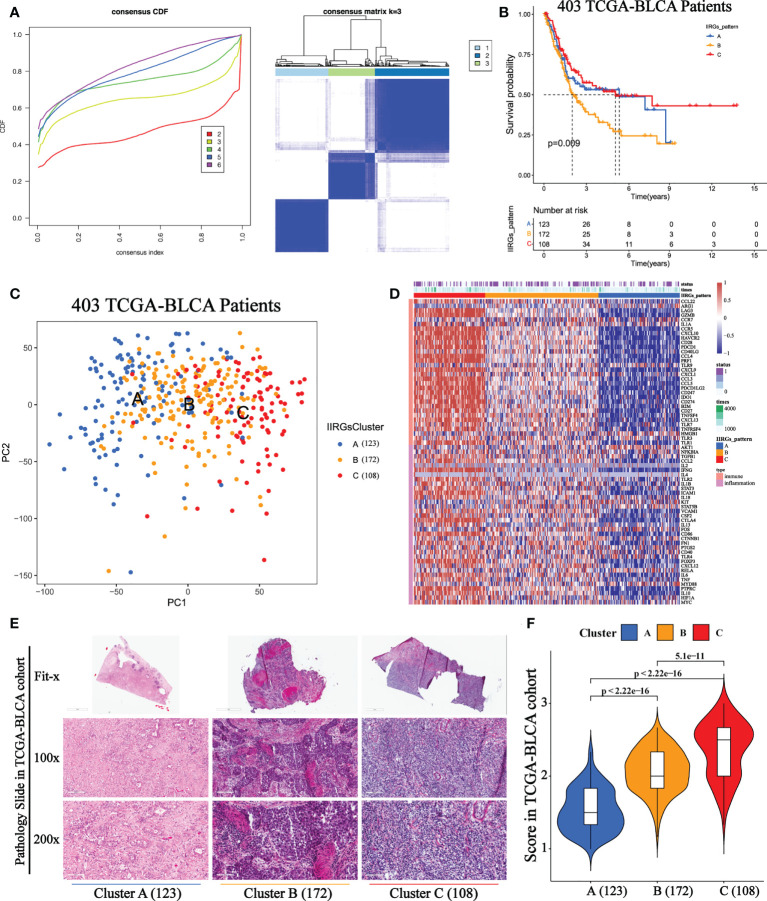
Unsupervised consensus cluster analysis of IIRGs in TCGA-BLCA cohort. **(A)** Heatmap of consensus clustering matrix for k =3 and Consensus clustering cumulative distribution function (CDF) for k = 2 to 6. **(B)** KM curves of OS among clusters in BLCA. **(C)** PCA among three IIRG modification patterns. **(D)** Hierarchical clustering of IIRGs in TCGA-BLCA cohort. **(E)** Representative photomicrographs of H&E staining of bladder sections among clusters. **(F)** The One-way ANOVA analysis of histopathological scores among clusters.

The optimal clustering division was three based on the minimum area under the CDF curve. The heatmap showed a relatively clear-cut boundary, indicating the reliability of clustering (**Figure 2A**). KM analysis revealed that patients in Clusters A and C had a survival benefit, while patients in cluster B had the worst outcome (**Figure 2B**). The PCA distribution patterns represented three different dynamic IIR types and validated three clusters assignment (**Figure 2C**). Moreover, the hierarchical clustering revealed that the expression of IIRGs among subtypes was significantly different, further verifying the reliability of clustering (**Figure 2D**). We further confirmed less immune and inflammatory cells infiltration in Cluster A and more infiltration in Cluster C (**Figures 2E, F**). Finally, two GEO cohorts were applied to prove the repeatability of classification (**Supplementary Figures 2A, B**).

### Existence of diverse tumor microenvironment infiltration among three identified patterns

The ESTIMATE and ssGSEA tools were used to analyze the levels of the immune score, stromal score, tumor purity, and inflammation score among clusters (**Supplementary Table 5**). Cluster A showed the highest tumor purity, while Cluster C referred to the highest inflammation, immune, and stromal scores, further validating the distribution of the three subclasses (**Supplementary Figures 3A-D**). Subsequently, we noticed that Cluster A showed the highest abundance of NK cells resting, T cells CD4 naïve, T cells regulatory (Tregs), and Plasma cells. On the other hand, cluster B was characterized by immature and resting immune cells, including Macrophages M0, Mast cells resting, and B cells naïve. This can help explain that it did not survive as well as Cluster A or Cluster C. Cluster C was primarily made up of activated immune cells, such as T cells CD8, T cells follicular helper, T cells CD4 memory activated, T cells CD4 memory resting, Neutrophils, and Macrophages M1 (**Supplementary Figure 3E**).

### Cluster B-specific differentially expressed immune cells and inflammatory activity might result in a poor outcome for TCGA-BLCA patients

**Figure 3:**
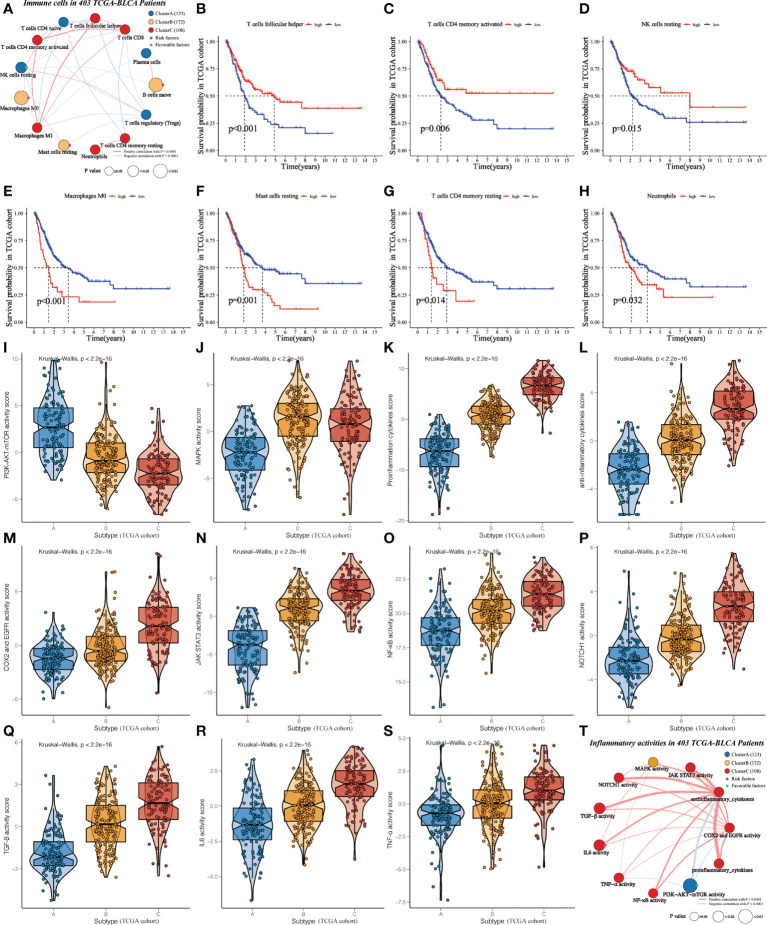
Identification of cluster-specific and prognosis-related differentially expressed immune cells and inflammatory activities in TCGA-BLCA cohort. **(A)** Interaction of cluster-specific and prognosis-related differentially expressed immune cells. Cluster A, blue; Cluster B, yellow; Cluster C, red. These circles highlighted in Green (purple) referred to favorable (risk) factors of overall survival. The size of each circle evaluated by Unicox p-values indicated the survival impact of each immune cell. The connection shows the interaction between two immune cells, and the thickness of the lines means the correlation strength among cells. The red (blue) lines represented the positive (negative) correlation. **(B-H)** KM curves were applied to estimate OS for the high- and low-level groups of differentially expressed immune cells. **(I-S)** The violin plots demonstrate the differences in expression levels of various inflammatory activities among clusters. **(T)** Interaction of cluster-specific and prognosis-related differentially expressed inflammatory activities.

In order to investigate the mechanisms leading to clinical phenotypic heterogeneity among clusters, univariate Cox regression was conducted to analyze 13 differentially expressed immune cells (**Supplementary Table 6**). Cluster A-specific NK cells resting were regarded as prognosis-related favorable factors (**Figure 3A**). While prognosis-related risk factors consisted of Cluster B-derived Macrophages M0, Mast cells resting, and B cells naïve. This might help explain the worst prognosis of cluster B despite having a high immune score. Further KM analysis showed that the high-level group of T cells follicular helper, T cells CD4 memory activated and NK cells resting had significantly higher survival probability than the low-level group (**Figures 3B–D**). Conversely, the abundance of Macrophages M0, Mast cells resting, T cells CD4 memory resting, and Neutrophils were related to poor prognosis (**Figures 3E–H**).

Furthermore, we assessed the relationship between clusters and inflammatory activity signatures. Cluster A positively correlated with the higher activity of PI3K-AKT-mTOR (**Figure 3I**), which was related to favorable prognosis (**Supplementary Figure 4A**). MAPK activity was regarded as the specific inflammatory activity of cluster B (**Figure 3J**). Cluster C showed the highest abundance of other inflammatory activities (**Figures 3K–S**), significantly related to OS (**Supplementary Figures 4B**-**3G**). Next, univariate Cox regression was conducted to analyze these differentially expressed inflammatory activities. PI3K-AKT-mTOR was cluster-specific favorable factor, which probably accounts for why Cluster A had a favorable prognosis (**Figure 3T**).

### Construction of the IIRS

**Figure 4:**
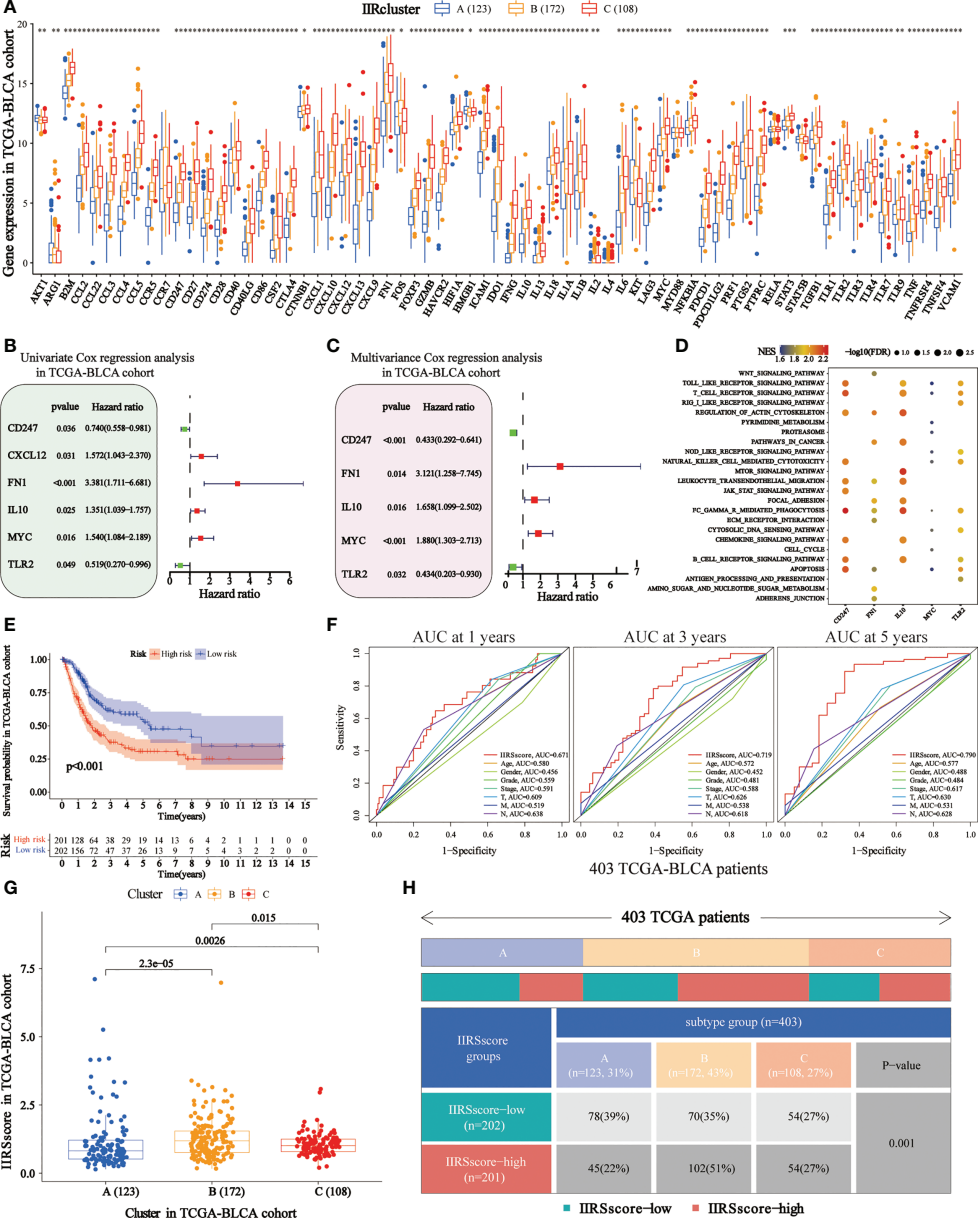
Construction of immune and inflammatory phenotype-related gene signature in TCGA-BLCA cohort. **(A)** Differences in the levels of the 85 IIRGs in three subtypes (***P< 0.001; **P< 0.01; *P< 0.05). **(B, C)** Forrest plot depicting the univariate and multivariance Cox regression analysis of DE. **(D)** Enrichment analysis for pathways in the high expression group of the IIRS genes. NES is the normalized enrichment score. **(E)** Survival analyses were applied to evaluate overall survival for the high- and low-risk groups in the TCGA train cohort. **(F)** AUCs (Area under ROC curve) for 1-, 3-, and 5-year OS and the risk score and clinical characteristics ROC curves. **(G)** Distributions of IIRS-score among clusters. **(H)** The Chi-square test was applied to evaluate the difference in immune and inflammatory patterns among patients in the high- and low-IIRS-score groups.

We obtained 62 DEIIRGs among clusters (**Figure 4A**). Univariate and multivariate Cox regression models identified 5 DEIIRGs (**Figures 4B, C**; **Supplementary Figure 4H**). The risk score = 1.13822603*Expression (FN1) + 0.50559931*Expression (IL10) + 0.631436*Expression (MYC) – 0.8375582*Expression (CD247) – 0.8336228*Expression (TLR2). GSEA further revealed that the five genes were correlated with immune pathways (**Figure 4D**).

### Genetic alteration of IIRS genes in TCGA-BLCA patients

The waterfall plot revealed 38 mutated DEIIRGs with higher than 0.5% mutation frequencies (**Supplementary Figures 5A, B**). Notably, TP53 and FN1 were regarded as the most frequently mutated DEIIRGs. Further pathway enrichment indicated that these 38 mutated DEIIRGs were significantly associated with the PD-L1 expression and PD-1 checkpoint pathway in cancer (**Supplementary Figure 5C**). Next, we unveiled the genetic alteration of IIRS genes. These genes are FN1 (5.34%), TLR2 (0.73%), IL10 (0.49%), MYC (0.49%), and CD247 (0.24%) (**Supplementary Figure 5D**).

### IIRS could predict OS and the IIR patterns

The KM analysis showed that the high IIRS-score group was significantly associated with low survival probability (**Figure 4E**). After adjusting clinical characteristics, 168 patients with complete clinical information were collected (**Supplementary Table 7**). The ROC curve revealed a high accuracy of IIRS in predicting the prognosis of BLCA patients, with an AUC of 0.671, 0.719, and 0.790 in 1, 3, and 5 years, respectively, compared to other clinical factors (**Figure 4F**). Compared to other models, IIRS showed the highest AUC in 5 years and a favorable predictive value in predicting 1- and 3- year OS (**Supplementary Figure 4I**), which revealed a favorable efficiency of our model in predicting short-term and long-term survival for BLCA patients.

Further, we noticed that patients in Cluster B showed a higher IIRS score than other subclasses (**Figure 4G**), which might help explain the survival inferiority of Cluster B. Simultaneously, patients in Cluster A showed a lower IIRS score compared to other molecular subclasses. Moreover, a Chi-square test demonstrated a significant difference in IIRS scores among IIR-clusters (**Figure 4H**). These results indicated that the IIRS had a strong predictive ability for survival prediction and IIR patterns.

### Two IIRS subtypes have distinct clinical behaviors

To investigate whether IIRS was related to OS among different clinical subgroups, the log-rank analysis indicated that lower risk scores were associated with favorable clinical characteristics in most subgroups (**Supplementary Figures 6A-E**).

### IIRS showed a good performance in predicting the clinical response of immunotherapy and chemotherapy

**Figure 5:**
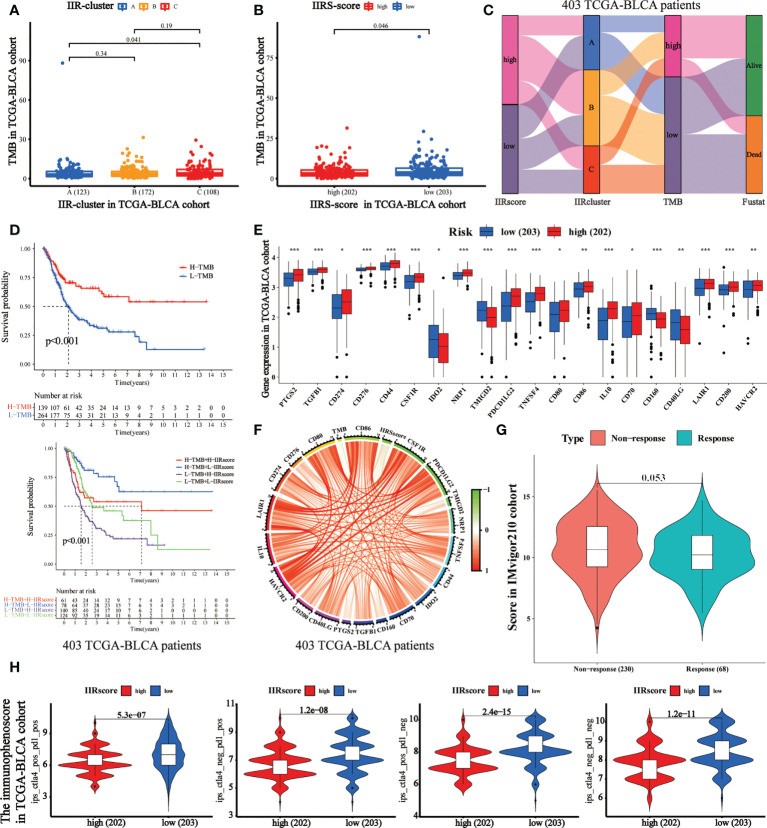
Evaluation of the performance of IIRS in the TCGA-BLCA patients’ immunotherapy. **(A)** The distribution of the TMB value in different IIR-clusters in TCGA-BLCA patients. **(B)** Differences in the TMB value between high- and low-risk IIRS groups. **(C)** The Sankey diagram was used to visualize the relation between IIR-clusters, IIRS-score groups, and TMB. Each column indicates a characteristic variable, different colors show different types, and lines represent the distribution of the same sample in different characteristic variables. **(D)** KM curves for different groups stratified by TMB or combining IIRS with TMB in the TCGA-BLCA cohort. **(E)** Differences in the expression of immune checkpoints between high- and low-risk IIRS groups. The upper and lower ends of the boxes refer to an interquartile range of values. **(F)** The correlation chord chart displays the mutual correlation among IIRS risk score, TMB, and checkpoints. **(G)** The differences in the clinical response (CR/PR, SD/PD) to anti–PD-1 immunotherapy in high or low IIRS-score groups in the IMvigor210 cohort. **(H)** The differences of the immunophenoscore (IPS) to anti–PD-1 or/and anti–CTLA4 immunotherapy in high or low IIRS-score groups. ***P < 0.001; **P < 0.01; *P < 0.05.

TMB has been regarded as an essential biomarker of the clinical response to immunotherapy in BLCA (24). We calculated the TMB value of each patient, and it was used to analyze the correlation between IIR clusters and IIRS scores (**Supplementary Table 8**). First, we noticed that patients in Cluster C (**Figure 5A**) and IIRS low-risk group (**Figure 5B**) showed the highest TMB values, matching their higher survival probability. The Sankey diagram further revealed that most Cluster B patients had high IIRS scores and low TMB, which was associated with a poor clinical outcome (**Figure 5C**). The further KM curve indicated that BLCA patients with high TMB showed a higher survival advantage than patients in the low TMB group (**Figure 5D**). Moreover, a high-TMB/low-IIRS risk score had the highest survival probability, whereas a low-TMB/high-IIRS score showed the worse clinical outcome (**Figure 5D**). Next, we noticed that almost all differentially expressed checkpoints were significantly upregulated in high IIRS-score groups compared with the low IIRS-score groups (**Figures 5E, F**).

Immunotherapies represented by PD-1 and CTLA-4 blockade have undoubtedly emerged as a critical breakthrough in BLCA therapy. We found that patients with low IIRS-score showed significant therapeutic advantages compared to those with high IIRS scores in the IMvigor210 cohort (**Figure 5G**). To further evaluate the relationship between IIRS and the clinical response to CTLA-4 and PD-1 blockers, we calculated the IPS of 402 BLCA patients. The results indicated that the low IIRS score was significantly correlated with CTLA-4 and PD-1 blockers (**Figure 5H**). Moreover, we noticed that the IC50 of potential majority drugs, including cisplatin, was significantly higher in the low IIRS-score group than in IIRS high-risk patients (**Supplementary Figure 7**).

### Evaluation of the IIRS genes’ expression

**Figure 6:**
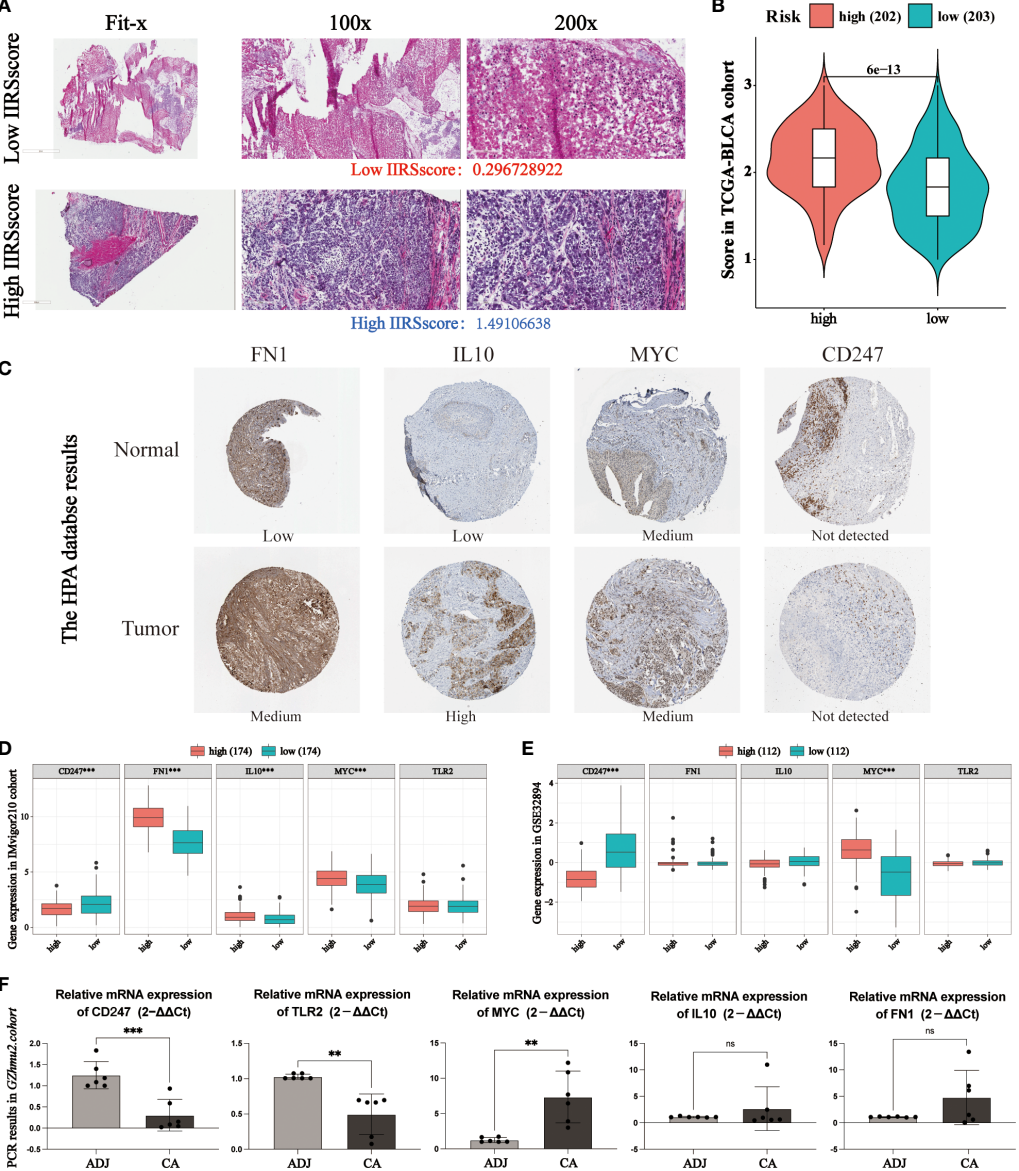
Evaluation of the expression of identified IIRS genes. **(A)** Representative photomicrographs of H&E staining of bladder sections between high or low IIRS-score groups. **(B)** Student’s T-test of histopathological scores between high or low IIRS-score groups. **(C)** The representative images of the immunohistochemical labeling of the identified genes of IIRGS on bladder tissues. Data were obtained from the human protein atlas (https://www.proteinatlas.org/). **(D, E)** Differences in the levels of the 5 identified genes of IIR signature in GEO cohorts. **(F)** Differences in the levels of the 5 identified genes of IIR signature in bladder cancer (CA) tissues and adjacent (ADJ) normal tissues. ***P < 0.001; **P < 0.01; ns refer to not statistically significant.

According to the histopathological section, the infiltration of immune and inflammatory cells in the high or low IIRS-risk group was significantly different (**Figures 6A, B**). We further explored the expression of five identified genes in the Human Protein Atlas (HPA) database. Compared with the normal tissues, FN1, IL10, and MYC were highly expressed, while the expression of CD247 was decreased in tumor bladder tissues (**Figure 6C**). Nevertheless, the HPA database has not included information on TLR2 due to its limited bladder tissues. So, we further evaluated the expression of the five proteins in two cohorts. In the IMvigor210 cohort, FN1, IL10, and MYC expressions in the IIRS high-risk group were significantly upregulated, while CD247 was significantly downregulated (**Figure 6D**). In the GSE32894 cohort, CD247 in the IIRS low-risk group was significantly upregulated, while MYC was significantly downregulated (**Figure 6E**). Finally, we evaluated the expressions of five identified genes in human bladders. As expected, MYC expressions in the cancer tissues were significantly upregulated, while CD247 and TLR2 were significantly downregulated in the adjacent normal tissues (**Figure 6F**). These data indicated that IIRS genes were differentially expressed between tumor tissues with corresponding normal or adjacent tissues.

### Evaluating the clinical application potential of the IIRS score

**Figure 7:**
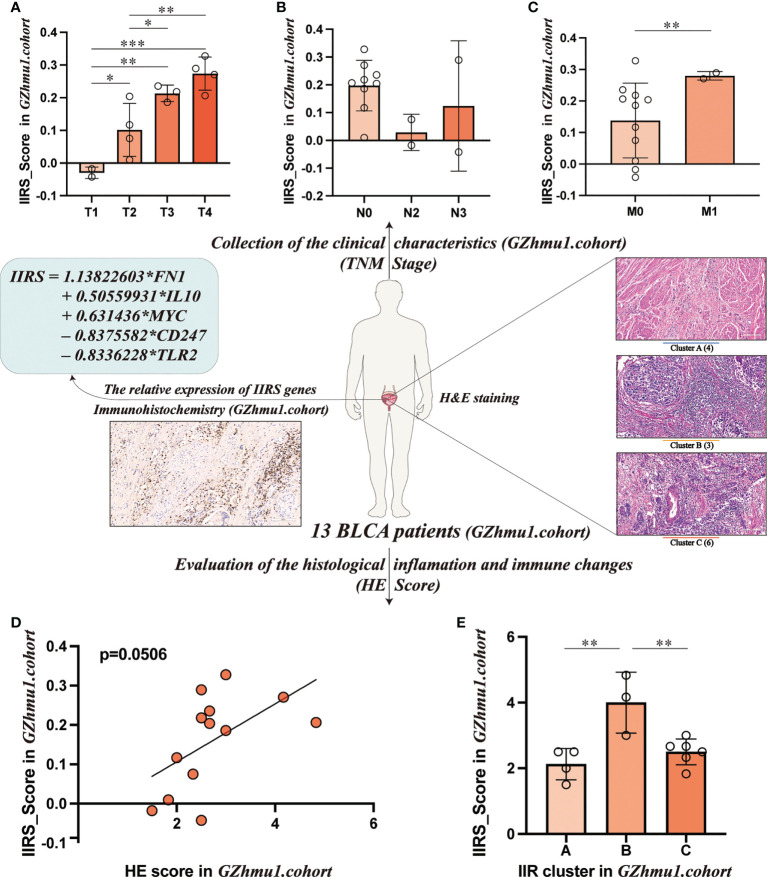
Evaluating the clinical application potential of IIRS-score in GZhmu1.cohort. **(A-C)** Differences in the IIRS-score among different TNM groups. **(D)** The correlation between IIRS-score and HE-score. **(E)** Distributions of IIRS-score among clusters. (***P< 0.001; **P< 0.01; *P< 0.05).

To evaluate the clinical application potential of the IIRS score, we first collected 13 bladder tissues of BLCA patients. First, the relative expression of five IIRS genes and the IIRS scores of 13 patients were evaluated by IHC (**Supplementary Table 9**). Intriguingly, these patients with higher T and M stages showed increased IIRS scores (**Figures 7A–C**). Next, the H&E-stained images evaluated the IIR cluster and inflammatory and immune scores of 13 patients (**Supplementary Table 10**). The HE-scores showed a nearly significant correlation with IIRS-scores (**Figure 7D**, p=0.0506), indicating that IIRS-score can effectively reflect the inflammatory/immune infiltration of bladder tissues. Furthermore, patients in Cluster B showed the highest IIRS scores than other subclasses (**Figure 7E**), which further proved that the IIRS score is a valid substitute for IIR patterns.

## Discussion

BLCA is one of the most frequent tumors in the urinary tract, with nearly 200,000 deaths reported worldwide annually, which carries a considerable financial and societal debt to countries (1, 2). Along with the growing molecular understanding of tumors, we have realized that BLCA is a heterogeneous disease with diverse oncogenic pathways and unique tumor microenvironment (TME) infiltration characteristics (2, 25). This molecular diversity bestows the poor treatment responses that oncologists often see when treating BLCA. Despite several therapeutic advances in BLCA, the traditional histological subtype usually fails to accurately predict clinical response to immunotherapy and chemotherapy (1, 5). Ever-increasing studies have revealed the critical role of immune and inflammatory responses (IIR) in facilitating or constraining the progression of tumors. Although several immune or inflammatory response-related patterns of BLCA have been identified (15–19), most of these researches seem to ignore the indivisible relationship between immune and inflammation. Thus, comprehensive analyses of IIR patterns in tumor microenvironment (TME) infiltration characteristics of BLCA remain urgently needed.

In the current study, three distinct and stable IIR subtypes have been identified and survival analysis suggested that cluster A and Cluster C had a favorable prognosis while cluster B had a worse prognosis. Previous studies demonstrated that normal controlled inflammation and immunity were correlated with favorable prognosis, while uncontrolled inflammation and immunity can result in disease progression (13, 26–29). Consistent with this, Cluster A and Cluster C may cause the normally controlled inflammation, while cluster B seems to be related to uncontrolled inflammation. H&E-stained histopathology images and immune scores confirmed clusters’ distinct infiltration of immune and inflammatory cells. A limited and balanced inflammation can initiate a favorable immune response (13, 26, 27), which is corroborated by the H&E results in cluster A. Patients in cluster C with a favorable prognosis showed massive infiltration of immune and inflammatory cells, which is in agreement with the concept that highly controllable inflammation in tumors is related to increased survival of patients with nearly any type of cancer (5, 27). However, some highly infiltrating cells may be retained in the stroma surrounding tumor nests, suggesting that these cells are ineffective in their duties as anti-tumor agents (5, 28). The stromal status, which is defined as “loose” or “dense,” might influence the migration of immune or inflammatory cells and restrict them from interacting with cancer cells (29). Following this, we observed that the high-level immune and inflammatory cells in cluster B were nearly nonexistent in the cancer cells, which might help explain the mismatched survival advantage of cluster B. We first identified cluster-specific and prognosis-related immune cells to further investigate the causes of these differences. Cluster A-specific NK cells resting were the prognosis-related favorable factors, while prognosis-related risk factors consisted of Cluster B-specific Macrophages M0, Mast cells resting, and B cells naïve. Although immature immune cells can help the body struggle with cancer, a recent study demonstrated that these immature cells could aid the metastatic spread, which helps explain the Cluster B-specific unfavorable prognosis (30, 31). Cluster C with a better prognosis was prominently composed of various activated immune cells, suggesting that they play a positive role in BLCA progression. Numerous activated function immune cells contribute to anti-tumor immunity, as they transmigrate across the stroma into the tumor (32).

Furthermore, we noticed that the relative abundance of 11 selected inflammatory activities differed significantly among clusters, suggesting the critical role of IIR in the development of BLCA. PI3K/AKT/mTOR and MAPK were activated in clusters A and B, respectively. Other inflammatory-activities populations were significantly more abundant in cluster C than in the other clusters, confirming the activated inflammatory status in cluster C. A growing number of researches have shown that inflammation plays a vital role in the progression of BLCA through various molecular basis and signaling pathways (14, 33–38). Cytokines, the most pivotal effector and messenger molecules in the immune and inflammatory responses, mediate the interactions between immune and non-immune cells in TME, thereby involving the occurrence, invasion, and migration of BLCA (14, 33). Cyclooxygenase-2 (Cox-2), a key enzyme that catalyzes the synthesis of prostaglandins, is overexpressed in BLCA and plays a pivotal role in inflammation-mediated stem cell proliferation/differentiation, thus promoting the growth of bladder tumors (34, 35). Moreover, the progression of BLCA involves alterations in multiple inflammation-related pathways that determine patients’ clinical characteristics and outcomes (5, 36–38). These changes were strongly associated with multiple cellular activities, such as cell proliferation, apoptosis, cycle progression, and angiogenesis. Overall, we observed the heterogeneity of the TME infiltration characteristics of BLCA among three IIR subtypes, which partially explains the distinct clinical outcome of patients among IIR subtypes. These results indicated that TME-associated immune and inflammatory infiltration plays a critical part in the progression of BLCA.

Given the specificity of IIR-modified phenotypes in individuals, we established a scoring model, IIRS, to evaluate the IIR modification pattern of individual patients with BLCA. Intriguingly, we noticed that patients in Cluster B showed the highest IIRS score with the worst prognosis compared to other subtypes, suggesting that the IIRS score was a dependable model for the comprehensive assessment of the IIR modification pattern and predicting the prognosis of patients. The IIRS was constructed based on five filtered genes: FN1, TLR2, IL10, MYC, and CD247. FN1, IL10, and MYC activation were related to the poor prognosis, indicating a pivotal role in cancer progression. FN1 was significantly elevated in several malignant tumors (39), which is in accordance with our results. Evolving lines of evidence have indicated that FN1 played a pivotal role in tumor cell proliferation, migration, invasion, and angiogenesis (39–41). As a crucial anti-inflammatory cytokine, IL10 has profound immunosuppressive functions, such as supporting immune escape and suppressing the expression of antigen-presenting cells, MHC-class II Ags, and costimulatory molecules on macrophages (42). Previous research has indicated that IL10 was reported to be upregulated in numerous advanced cancers (43), confirmed by the present work. MYC activation has been reported to regulate the expression of several immune checkpoints, inactivate macrophages and DCs, and limit NK and T cells (44, 45). The inactivation of MYC triggers tumor regression through the loss of hallmark features of cancer (45). The upregulation of TLR2 and CD247 in current work was significantly associated with the superior prognosis, suggesting that TLR2 and CD247 activation may be essential for anti-tumor immunity. In agreement with our concept, TLR2 was reported to initiate the innate and sustained adaptive immune responses in cancer (46, 47). Moreover, the downregulation of CD247 in T cells was associated with immunosuppression due to chronic inflammation (11). Taken together, previous research and our results indicated that the IIRS genes might act as potential biomarkers and therapeutic targets for BLCA.

In the last decade, the accumulated interest in immunotherapy and chemotherapy, coupled with a growing understanding of the pathogenesis of BLCA, has dramatically enriched the therapeutic choices against BLCA (1, 3). Accumulating evidence has revealed that TMB can enable oncologists to identify patients who are likely to benefit from immunotherapy (24), which is in line with our finding that high TMB has a significant correlation with favorable prognosis. Additionally, checkpoint blockades significantly impact cancer immunotherapy (48). Preliminary pathway annotation analysis indicated that mutated DEIIRGs were significantly associated with the PD-L1 expression and PD-1 checkpoint pathway in cancer. It was also found that the vast majority of checkpoint molecules were negatively correlated to IIRS-score, indicating that IIRS may play a pivotal role in immunotherapeutic response prediction. With the IMvigor210 cohort and TCIA database, IIRS was further verified to be efficient in predicting the response to immunotherapy. Considering that traditional cisplatin-based chemotherapy still plays a non-negligible role in the personalized medicine era (3), we assessed the IC50 of potential drugs against BLCA. Patients with low IIRS-score were more sensitive to most potential drugs than the high IIRS-score group, suggesting that the low IIRS-score group was more likely to benefit from chemotherapy. Although our analyses provided different therapeutic options for high and low IIRS-score groups, the efficacy and mechanism of these drugs against BLCA still require further demonstration.

The present work is the first to establish the immune and inflammatory phenotype-related prognosis, immunotherapy, and chemotherapy signature for BLCA. Intriguingly, we observed that IIRS could even predict the prognosis of BLCA patients with different subclasses stratified by clinical traits. Moreover, they may effectively improve the prediction of prognosis when compared to conventional staging. To our knowledge, the five-gene prognostic signature described herein has not been reported previously. The current study has certain limitations that deserve mention. On the one hand, this work was a retrospective design with heterogeneity due to comparisons between patients from cross-platform data. A comprehensive prospective study is even more necessary to affirm the complete prediction ability of the IIRS. On the other hand, potential driver molecules in our research require further functional validation, and their detailed molecular mechanisms in the pathogenesis of BLCA need further elucidation.

## Data availability statement

Publicly available datasets were used in this study, the names of the repositories/accession numbers are in the article/**Supplementary Materials**.

## Author contributions

ZC: Conceptualization, Investigation, Writing - Original Draft. RL: Writing - Review and Editing. JZ: Validation. LA: Writing - Review and Editing. GZ: Formal analysis. ML: Visualization. JD: Investigation. RY: Formal analysis. ZS: Visualization. WZ: Funding acquisition. DQ: Funding acquisition. XD: Supervision. SL: Supervision. BS: Supervision. WW: Project administration. All authors contributed to the article and approved the submitted version.

## Funding

This work was supported by grants from The National Natural Science Foundation of China (No.61931024), and The Science and Technology Plan Project of Guangzhou (Grant NO. 201804020023).

## Acknowledgments

We thank Home for Researchers editorial team (www.home-for-researchers.com) for language editing service.

## Conflict of interest

The authors declare that the research was conducted in the absence of any commercial or financial relationships that could be construed as a potential conflict of interest.

## Publisher’s note

All claims expressed in this article are solely those of the authors and do not necessarily represent those of their affiliated organizations, or those of the publisher, the editors and the reviewers. Any product that may be evaluated in this article, or claim that may be made by its manufacturer, is not guaranteed or endorsed by the publisher.

## Supplementary material

Supplementary Figure 1 Collection of Inflammation-Immunity-related genes. **(A)** Screening out the Inflammation-related union genes using six gene sets. **(B)** The “Immune Cell-Target” network. **(C)** Identification of Inflammation-related core genes. **(D)** Identification of Immune-related core genes after screening. **(E)** The Venn diagram of drug-disease crossover genes.

Supplementary Figure 2 Validation of three IIR patterns in two GEO cohorts. **(A, B)** Hierarchical clustering of IIRGs in GEO cohorts and the KM curves of OS for three IIRGs modification patterns.

Supplementary Figure 3 Comparison of composition and immune cells infiltration of TME among three subtypes in TCGA-BLCA cohort. **(A-C)** Comparison of the composition of TME (immune score, stromal score, and tumor purity) among three subgroups. **(D)** Comparison of inflammation score among three subgroups. **(I)** Differences in the abundances of the 22 immune cells infiltration in IIRGs subtypes.

Supplementary Figure 4
**(A-G)** KM curves were applied to estimate overall survival for the high- and low-level groups of differentially expressed immune cells. **(H)**The interaction of prognosis-related differentially expressed IIRGs. **(I)** The AUC values were applied to compare models’ differences in predicting 1-, 3-, and 5-year OS.

Supplementary Figure 5 Tumor mutation analysis of DEIIRGs in TCGA-BLCA cohort. **(A)** The mutation profile of DEIIRGs in TCGA-BLCA patients. **(B)** A visual summary of these mutated genes is displayed as a tag cloud, where more frequent genes are displayed using a larger font size. **(C)** Enrichment analysis of KEGG signal pathway of the mutated DEIIRGs with mutation frequencies higher than 1%. **(D)** Lollipop plot displaying mutation distribution and protein domains for identified genes in cancer with the labeled recurrent hotspots.

Supplementary Figure 6 Evaluation of the performance of IIRS in different clinical subgroups of TCGA-BLCA patients. **(A-E)** The KM curves of IIRS in BLCA patients with different ages, gender, stage, T, and N.

Supplementary Figure 7 Chemotherapeutic responses of high- and low-risk TCGA-BLCA patients. **(A, B)** The box plots of the estimated IC50 for selected drugs.
