# Supplementary figures and images for: Distinct immune and inflammatory response patterns contribute to the identification of poor prognosis and advanced clinical characters in bladder cancer patients
Source: Front Immunol. 2022 Oct 27;13:1008865. doi: 10.3389/fimmu.2022.1008865 (PMC9646535; doi:10.3389/fimmu.2022.1008865)

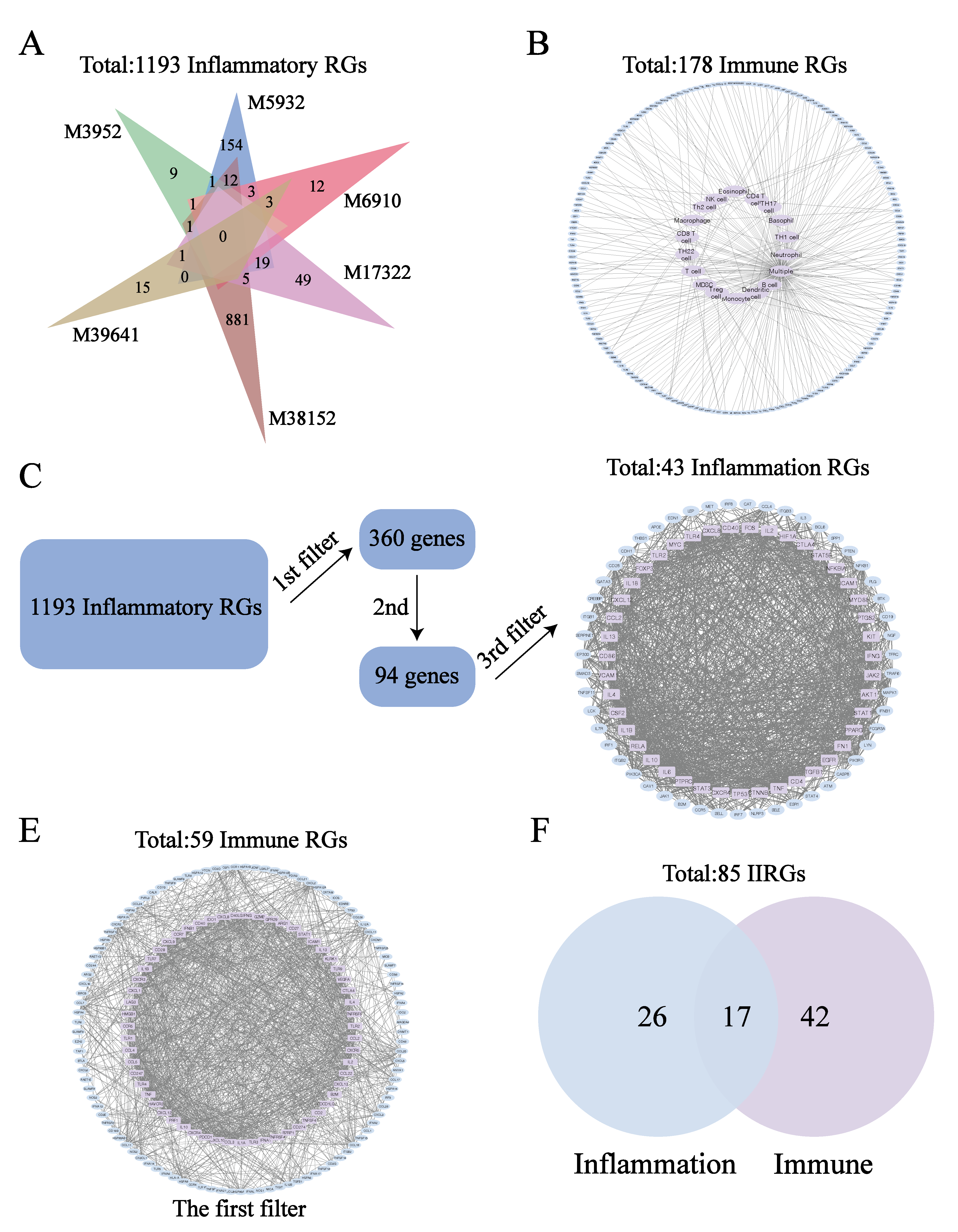

Supplement: Supplementary Figure 1 — Collection of Inflammation-Immunity-related genes. (A) Screening out the Inflammation-related union genes using six gene sets. (B) The “Immune Cell-Target” network. (C) Identification of Inflammation-related core genes. (D) Identification of Immune-related core genes after screening. (E) The Venn diagram of drug-disease crossover genes. [file Image_1.png]

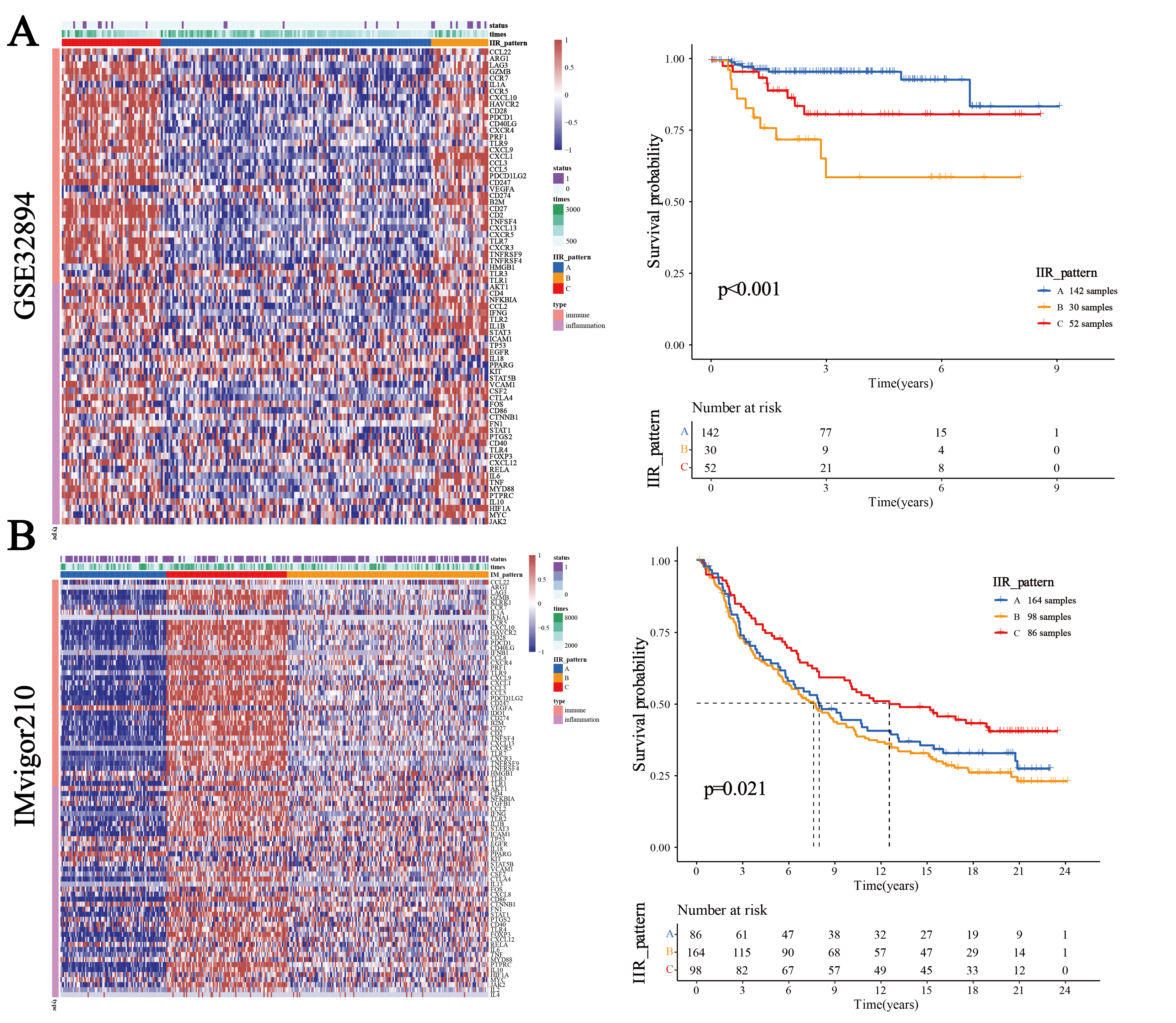

Supplement: Supplementary Figure 2 — Validation of three IIR patterns in two GEO cohorts. (A, B) Hierarchical clustering of IIRGs in GEO cohorts and the KM curves of OS for three IIRGs modification patterns. [file Image_2.png]

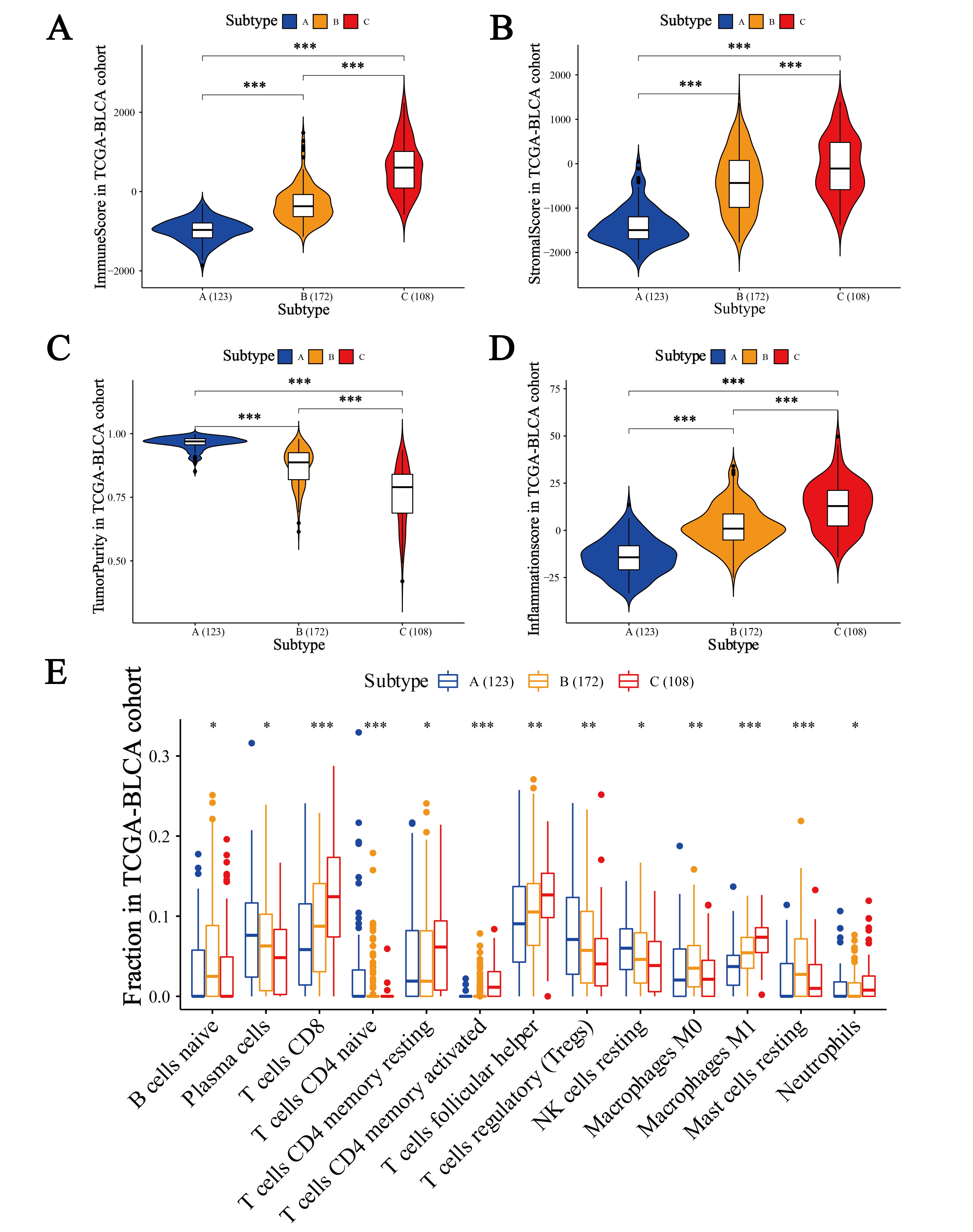

Supplement: Supplementary Figure 3 — Comparison of composition and immune cells infiltration of TME among three subtypes in TCGA-BLCA cohort. (A-C) Comparison of the composition of TME (immune score, stromal score, and tumor purity) among three subgroups. (D) Comparison of inflammation score among three subgroups. (I) Differences in the abundances of the 22 immune cells infiltration in IIRGs subtypes. [file Image_3.png]

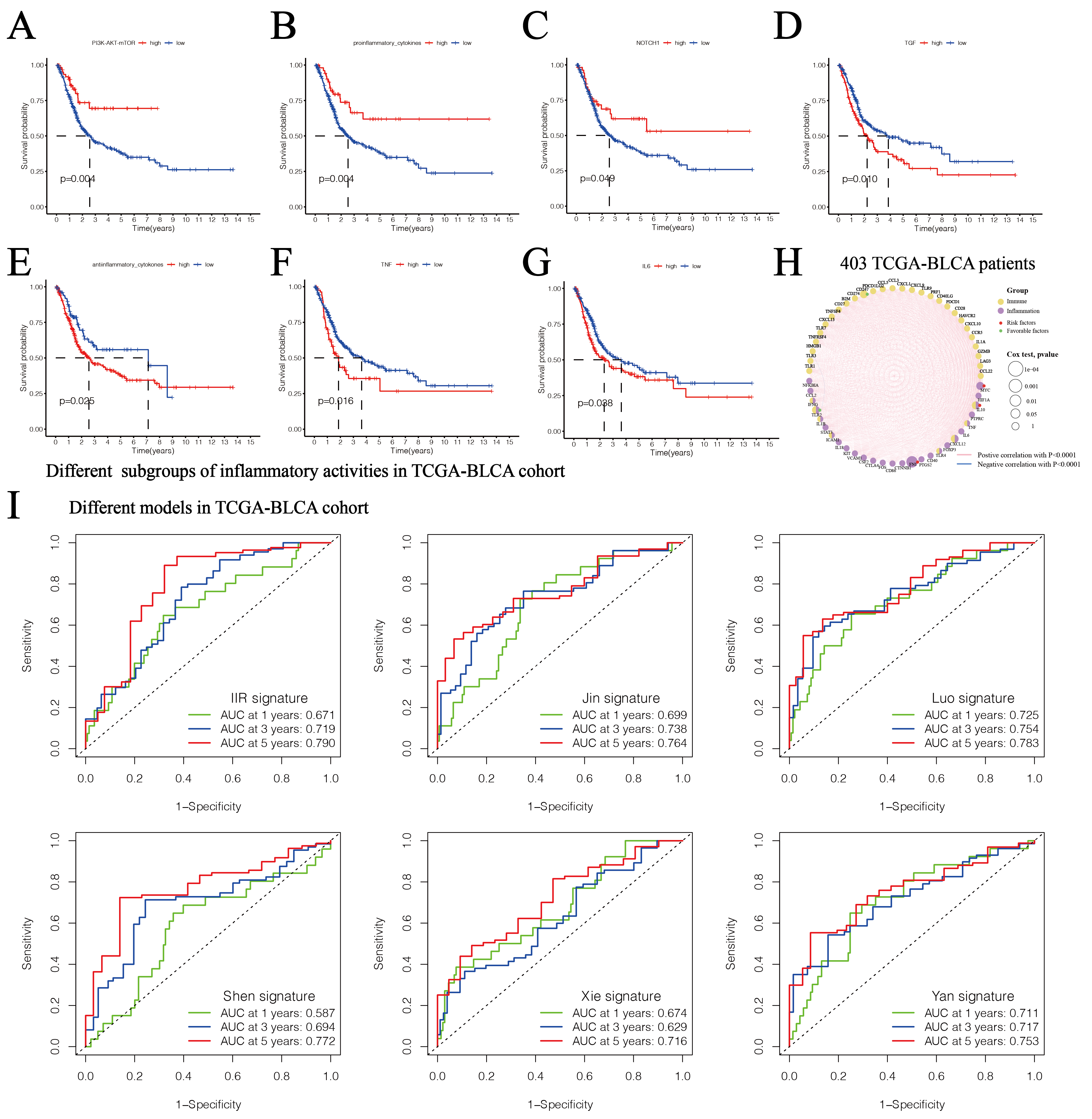

Supplement: Supplementary Figure 4 — (A-G) KM curves were applied to estimate overall survival for the high- and low-level groups of differentially expressed immune cells. (H)The interaction of prognosis-related differentially expressed IIRGs. (I) The AUC values were applied to compare models’ differences in predicting 1-, 3-, and 5-year OS. [file Image_4.png]

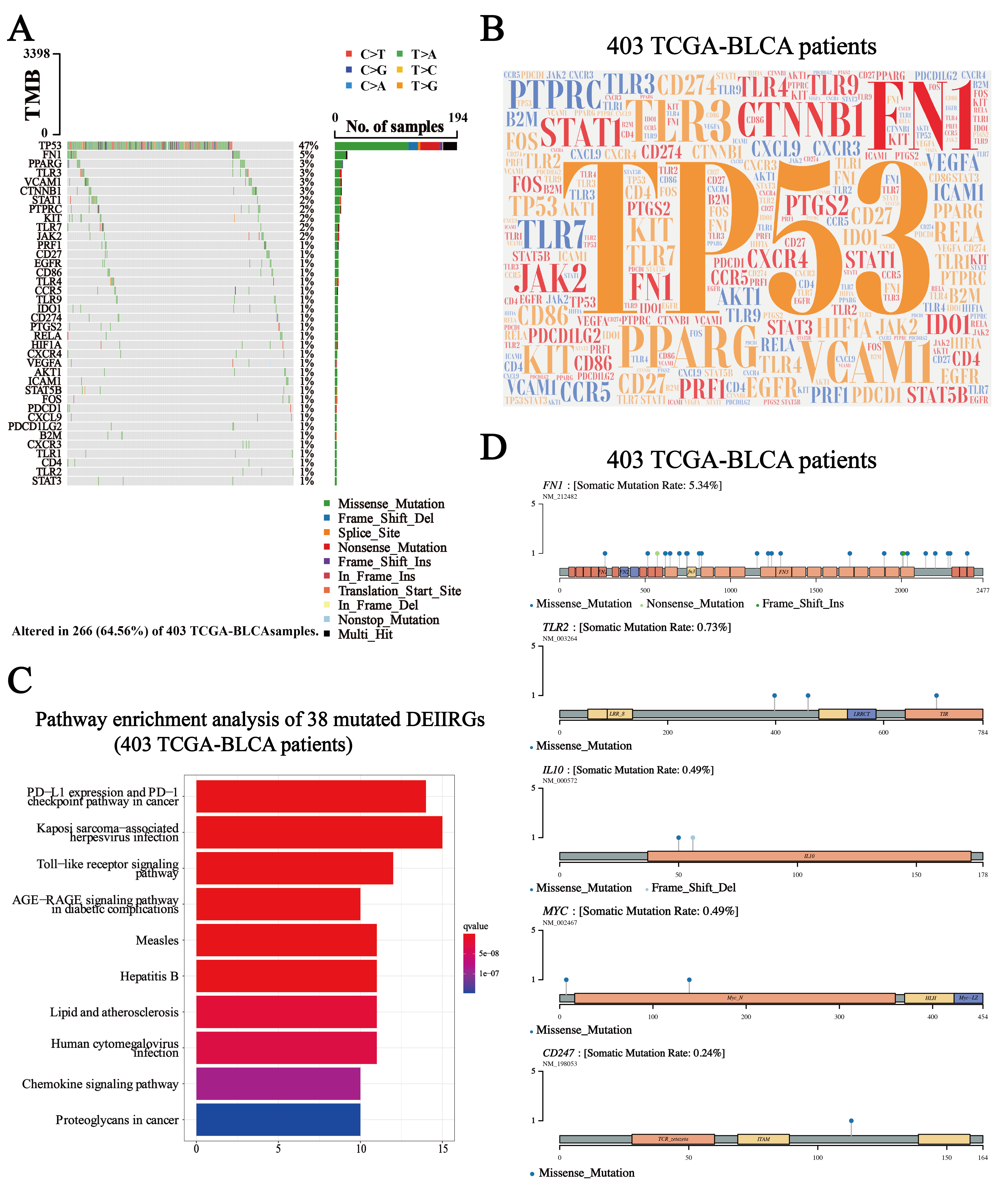

Supplement: Supplementary Figure 5 — Tumor mutation analysis of DEIIRGs in TCGA-BLCA cohort. (A) The mutation profile of DEIIRGs in TCGA-BLCA patients. (B) A visual summary of these mutated genes is displayed as a tag cloud, where more frequent genes are displayed using a larger font size. (C) Enrichment analysis of KEGG signal pathway of the mutated DEIIRGs with mutation frequencies higher than 1%. (D) Lollipop plot displaying mutation distribution and protein domains for identified genes in cancer with the labeled recurrent hotspots. [file Image_5.png]

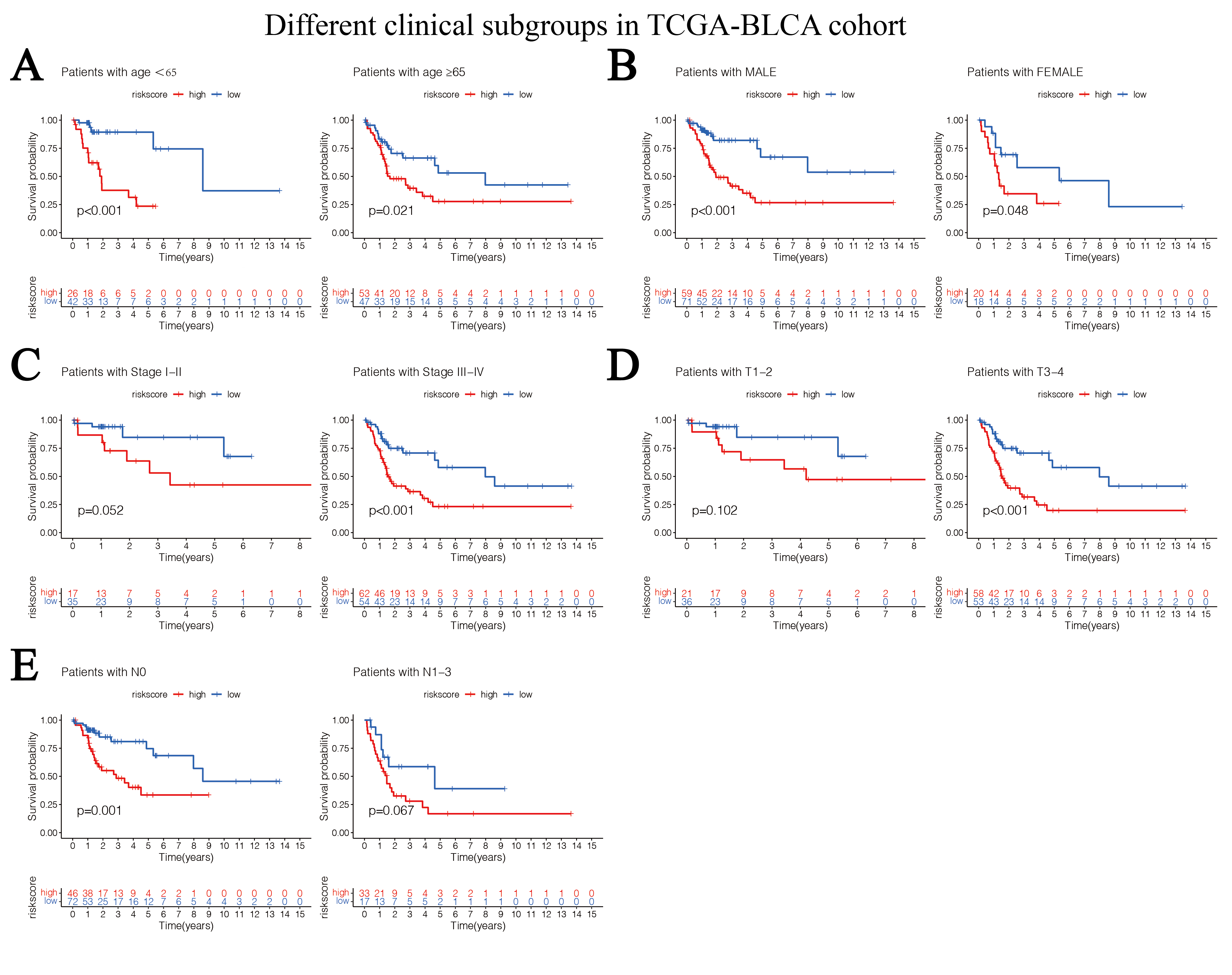

Supplement: Supplementary Figure 6 — Evaluation of the performance of IIRS in different clinical subgroups of TCGA-BLCA patients. (A-E) The KM curves of IIRS in BLCA patients with different ages, gender, stage, T, and N. [file Image_6.png]

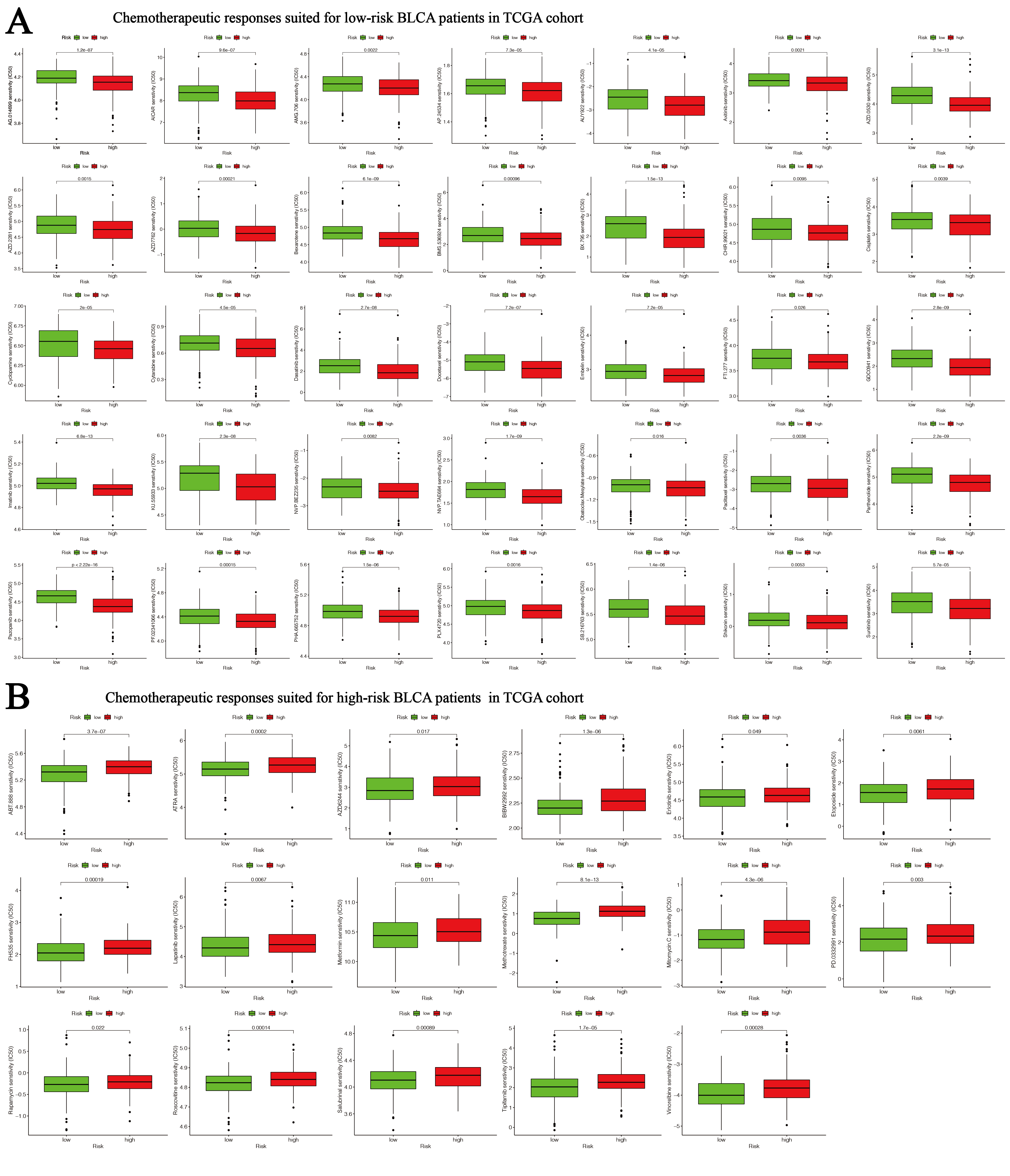

Supplement: Supplementary Figure 7 — Chemotherapeutic responses of high- and low-risk TCGA-BLCA patients. (A, B) The box plots of the estimated IC50 for selected drugs. [file Image_7.png]
